# Progress Report on EMBED: A Pragmatic Trial of User-Centered Clinical Decision Support to Implement EMergency Department-Initiated BuprenorphinE for Opioid Use Disorder

**DOI:** 10.20900/jpbs.20200003

**Published:** 2020-02-21

**Authors:** Edward R. Melnick, Bidisha Nath, Osama M. Ahmed, Cynthia Brandt, David Chartash, James D. Dziura, Erik P. Hess, Wesley C. Holland, Jason A. Hoppe, Molly M. Jeffery, Liliya Katsovich, Fangyong Li, Charles C. Lu, Kaitlin Maciejewski, Matthew Maleska, Jodi A. Mao, Shara Martel, Sean Michael, Hyung Paek, Mehul D. Patel, Timothy F. Platts-Mills, Haseena Rajeevan, Jessica M. Ray, Rachel M. Skains, William E. Soares, Ashley Deutsch, Yauheni Solad, Gail D’Onofrio

**Affiliations:** 1 Department of Emergency Medicine, Yale University School of Medicine, New Haven, CT 06519, USA; 2 Yale University School of Medicine, New Haven, CT 06510, USA; 3 Yale Center for Medical Informatics, Yale University School of Medicine, New Haven, CT 06511, USA; 4 Yale Center for Analytical Sciences, Yale School of Public Health, New Haven, CT 06520, USA; 5 Department of Emergency Medicine, University of Alabama at Birmingham School of Medicine, Birmingham, AL 35233, USA; 6 Department of Emergency Medicine, University of Colorado, Aurora, CO 80045, USA; 7 Department of Emergency Medicine, Mayo Clinic, Rochester, MN 55905, USA; 8 The Patient Revolution, New Haven, CT 06511, USA; 9 Information Technology Services, Yale New-Haven Health, New Haven, CT 06519, USA; 10 Department of Emergency Medicine, University of North Carolina School of Medicine, Chapel Hill, NC 27599, USA; 11 Department of Emergency Medicine, University of Massachusetts Medical School-Baystate, Baystate, MA 01199, USA

**Keywords:** user-centered design, clinical decision support systems, opioid-related disorders, opioid use disorder, buprenorphine, health information technology, electronic health records, health informatics, quality improvement, medication for opioid use disorder (MOUD)

## Abstract

Buprenorphine (BUP) can safely and effectively reduce craving, overdose, and mortality rates in people with opioid use disorder (OUD). However, adoption of ED-initiation of BUP has been slow partly due to physician perception this practice is too complex and disruptive. We report progress of the ongoing EMBED (*EMergency department-initiated BuprenorphinE for opioid use Disorder)* project. This project is a five-year collaboration across five healthcare systems with the goal to develop, integrate, study, and disseminate user-centered Clinical Decision Support (CDS) to promote the adoption of Emergency Department (ED)-initiation of buprenorphine/naloxone (BUP) into routine emergency care. Soon to enter its third year, the project has already completed multiple milestones to achieve its goals including (1) user-centered design of the CDS prototype, (2) integration of the CDS into an automated electronic health record (EHR) workflow, (3) data coordination including derivation and validation of an EHR-based computable phenotype, (4) meeting all ethical and regulatory requirements to achieve a waiver of informed consent, (5) pilot testing of the intervention at a single site, and (6) launching a parallel group-randomized 18-month pragmatic trial in 20 EDs across 5 healthcare systems. Pilot testing of the intervention in a single ED was associated with increased rates of ED-initiated BUP and naloxone prescribing and a doubling of the number of unique physicians adopting the practice. The ongoing multi-center pragmatic trial will assess the intervention’s effectiveness, scalability, and generalizability with a goal to shift the emergency care paradigm for OUD towards early identification and treatment.

**Trial Registration:**

Clinicaltrials.gov # NCT03658642.

## INTRODUCTION

The opioid epidemic is a public health crisis that has devastated countless families and communities in the United States [1,2]. As of 2018, approximately 2 million people in the US had been diagnosed with opioid use disorder (OUD) [3] and more than 33,000 individuals die annually from opioid-related causes [4]. The Emergency Department (ED) serves as a major access point to medical care for patients with OUD. In 2011, there were 605,000 opioid-related emergency department (ED) visits [5]. Also, there was a 30% increase in ED visits for opioid overdose between 2016 and 2017 [6]. Of 11,557 ED patients in Massachusetts who were treated for a non-fatal opioid overdose, 5.5% died within 1 year, 1.1% within 1 month, and 0.25% within 2 days [7]. As such, patients discharged from the ED following a non-fatal opioid overdose face a high short-term risk of death. Therefore, emergency clinicians have a unique opportunity to initiate addiction treatment that could prevent a subsequent fatal overdose. However, emergency clinicians have historically provided treatment for the immediate complication of addiction that prompted the ED visit, relying on community-based opioid treatment programs (OTPs) to initiate MOUD) [8].

The U.S. Food and Drug Administration (FDA) has approved three medications for OUD (MOUDs): methadone, buprenorphine, and naltrexone. These medications have shown consistent benefits across many outcomes; in particular, buprenorphine has a profile amenable to treatment initiation in the ED. Buprenorphine/naloxone (BUP), a combination of partial opioid agonist and an antagonist, can effectively reduce withdrawal symptoms, craving, and opioid use, with corresponding decreases in mortality (both all-cause and opioid-related) [9-12]. A systematic review of 31 randomized controlled trials (5430 participants) comparing BUP maintenance treatment versus placebo or methadone in the management of OUDs, found buprenorphine to be an effective medication in maintenance treatment [11]. In a randomized placebo-controlled trial in Sweden of 40 OUD patients comparing cognitive behavioral therapy plus daily BUP or opioid tapering followed by placebo, demonstrated that retention at one-year was 75% with BUP compared to 0% with placebo [12]. Furthermore, in a two-phase randomized controlled trial in 653 treatment-seeking outpatients with OUD treated with BUP, no difference was found in successful outcomes based on adjunctive counseling intensity [13]. However, despite such evidence for the effectiveness of BUP, less than one-third (28.5%) of the 2 million Americans currently with OUD receive some form of MOUD [14]. A 2015 randomized clinical trial of 329 opioid-dependent patients who were treated at an urban teaching hospital ED demonstrated both that BUP initiation in the ED is safe and that ED patients with untreated OUD receiving BUP in the ED are twice as likely to remain engaged in addiction treatment at one month (78% vs 37%, *p* < 0.001) [14]. Despite this evidence, adoption of this evidence-based practice has been slow [15-17].

Several logistical and regulatory barriers have contributed to the slow adoption of BUP initiation into routine emergency practice, including lack of physician familiarity with the BUP care algorithm, physician perception that the BUP initiation workflow is too complicated and disruptive to the ED environment [18]. Furthermore, the poor usability of current electronic health records (EHRs) makes it difficult to streamline unfamiliar practices like this in the complex and dynamic ED context [19]. To address these barriers and facilitate adoption of ED BUP initiation, the EMBED (EMergency department-initiated BuprenorphinE for opioid use Disorder) project aims to develop, integrate, study, and disseminate user-centered clinical decision support (CDS) tools to promote the adoption of ED BUP initiation into routine emergency care. In this report, we present our progress toward accomplishing these goals, including: (1) designing the user-centered CDS, (2) integrating the CDS into an automated electronic health record (EHR) workflow, (3) derivating and validating an EHR-based computable phenotype to identify potentially eligible patients, (4) meeting all ethical and regulatory requirements to achieve a waiver of informed consent, (5) pilot testing the intervention at a single site, and (6) launching a parallel group-randomized trial in 20 EDs across 5 healthcare systems to evaluate the effectiveness and scalability of the intervention.

## METHODS

### Study Aims

The project was proposed to be implemented in two phases - the planning phase (year 1) and the trial phase (years 2–5). Aim 1 of the planning phase was to develop a user-centered, EHR-based CDS tool, that would (1) identify adult ED patients potentially eligible for BUP initiation, (2) facilitate emergency clinician decision-making around BUP initiation in the ED, and (3) generate automatic referral for ongoing MOUD at community-based treatment sites. Aim 2 of the planning phase was to establish the infrastructure for the proposed trial. For the implementation phase, Aim 1 was to test the intervention in an 18-month pragmatic, cluster randomized trial in 20 EDs across five healthcare systems: (1) Yale-New Haven Health, (2) University of North Carolina (UNC) Health Care, (3) University of Alabama, Birmingham, (4) University of Colorado, and (5) University of Massachusetts-Baystate. Aim 2 for the implementation phase is to disseminate the trial findings nationally.

The overall goal of this pragmatic trial is to evaluate the effectiveness of the proposed intervention to increase the rate of BUP administration in the ED for OUD patients compared to usual ED care. Our long-term goal is to accelerate wide-scale adoption of the practice of ED initiation of BUP for OUD patients into routine emergency care nationally.

### Study Design and Randomization

The study design for this pragmatic trial is an 18-month, parallel, cluster-randomised, superiority trial using constrained randomization of clusters to arms. The unit of randomization (i.e., cluster) is the ED. Twenty ED sites across five healthcare systems were allocated 1:1 to CDS or Intervention Site (with user-centered CDS integrated into the EHR) and Control Site/Usual Care groups using stratified covariate constrained randomization. The variables included in the constrained randomization procedure included: (1) EHR vendor, (2) community or academic, (3) annual patient volume (visits/year), (4) annual patient volume meeting EHR phenotype, (5) ongoing OUD resources, and (6) proportion of Drug Addiction Treatment Act of 2000 (DATA 2000) waivered attending physicians (required to prescribe BUP). Stratification of the constrained randomization occurred by healthcare system to control for potential system differences.

### Participants

Study participants include all ED attending physicians practicing in study site EDs. The analysis will include those physicians’ encounters with patients who meet an EHR-based computable phenotype suggesting possible OUD and who are not currently engaged in MOUD treatment. Only encounters with discharged, non-pregnant ED patients aged 18 years or older who will be included in the analysis.

### Intervention

The study intervention is the user-centered CDS developed in the planning phase of this study, along with brief just-in-time user training to teach emergency clinicians how to use the intervention). Full details of the CDS intervention are reported in the Results section as an outcome of the planning phase. EDs not randomized to the intervention will provide usual care.

### Outcomes

The primary outcome of the trial is the proportion of included patient encounters that result in either BUP initiation in the ED or an ED prescription for BUP. Secondary outcomes include the proportion of patient encounters that result in (1) referral for ongoing MOUD treatment and (2) prescription of naloxone at discharge from the ED. Other secondary outcomes measure the proportion of attending physicians who (3) provided any ED-initiated BUP during the trial period and (4) received a DATA 2000 waiver to prescribe BUP. We *hypothesize* that rate of ED-initiation of BUP will be higher in EDs included in the intervention arm of the trial.

### Data Collection, Coordination, and Validation

With the exception of some physician-level outcomes (e.g., the proportion of attendings with DATA 2000 waivers), all trial data will be collected from clinical data entered in the EHR. No protected health information (PHI) will be collected, including the date of service; instead, the elapsed number of days since the trial launched at each site will be shared with the investigative team who are also blinded to the exact launch dates. All study variables and outcomes have been mapped to a *master data dictionary* with a corresponding *data specification* document that includes all requirements for data formatting and coordination. Each healthcare system has assembled a *structured query language (SQL) query* for data retrieval from their local EHR databases. To ensure that each system’s data set matched the final study variables, each query was validated by generating sample data sets from each system. Three rounds of sample data review have been conducted. Each system also reviewed 10 randomly selected charts to ensure all study data collected from the EHR had *face validity* with the corresponding clinical data and the data dictionary. An *online data portal* was created to coordinate all trial data submission. Data collection is underway at all study sites with monthly uploads to the data portal. Data will be monitored by the study data coordination team at monthly intervals for completeness.

### Analysis Plans

Analyses of primary and secondary outcomes will be conducted using logistic regression with weighted generalized estimating equations (GEE) to account for clustering from the EDs and physicians in patient-visit-level outcome models (physician-level models for outcomes 3 and 4 above). Analyses will be conducted as intention-to-treat, including all individuals regardless of intervention use (i.e., we will count visits whether or not the CDS was used). While the unit of randomization is at the level of the ED, the unit of analysis will be the patient or attending physician. For patient-level analyses, only the first ED encounter for an individual patient will be used. Sensitivity analyses will include all ED visits, including repeat visits.

### Ethics and Dissemination

The protocol has been approved by the Western Institutional Review Board (WIRB); all five collaborating healthcare systems have implemented reliance agreements between the local IRB and WIRB. A waiver of individual informed consent was obtained, with the following reasoning: (1) the intervention was deemed minimal risk since no identifiers will be collected and no proposed treatments are experimental; (2) patients retain the right to refuse treatment or request treatment at any time; (3) all components of the CDS intervention are validated clinical tools that are part of recommended best practices; (4) both intervention and non-intervention site clinicians retain all control of their practice: intervention site clinicians are not required to use the CDS or initiate BUP in any patients, while non-intervention site clinicians have access to all standard OUD medications and services to which they would otherwise have access in the absence of the study. To that end, patient- and provider-facing posters detailing the study objectives and the care algorithm for ED BUP initiation are posted at all study sites regardless of intervention or control status. An Independent Study Monitor will be utilized in place of a Data Safety Monitoring Board. Results will be reported in ClinicalTrials.gov (NCT03658642) and published in open-access, peer reviewed journals, presented at national meetings, and shared with the clinicians at participating sites via a broadcast email notification of publications. After the trial ends, we will widely disseminate the study findings and best practices around ED-initiation of BUP for OUD patients, as well as share our software and other relevant computer code to make the intervention freely available.

Full details of the study protocol are available in a May 2019 BMJ 9 BMJ Open publication by Melnick et al. [20].

## RESULTS

### User-Centered Design of the CDS Prototype

The intervention was designed and formatively evaluated following a user-centered design approach with iterative prototype development in 5 phases that included 26 attending and resident physician users. Through initial observations and interviews of ED physicians, we were able to identify varying levels of familiarity with the ED BUP initiation protocol and varying support needs across the user experience. This led to the development of a flexible design offering direct care pathways for more experienced users as well as additional, optional decision support for less-experienced users needing assistance with assessing for OUD, evaluating withdrawal severity, and motivating patients’ readiness to start treatment. Full details of the user-centered design process were published by Ray et al. in JMIR Human Factors in February 2019 [21].

### Derivation and Validation of an EHR-Based Computable Phenotype

**Figure 1.**
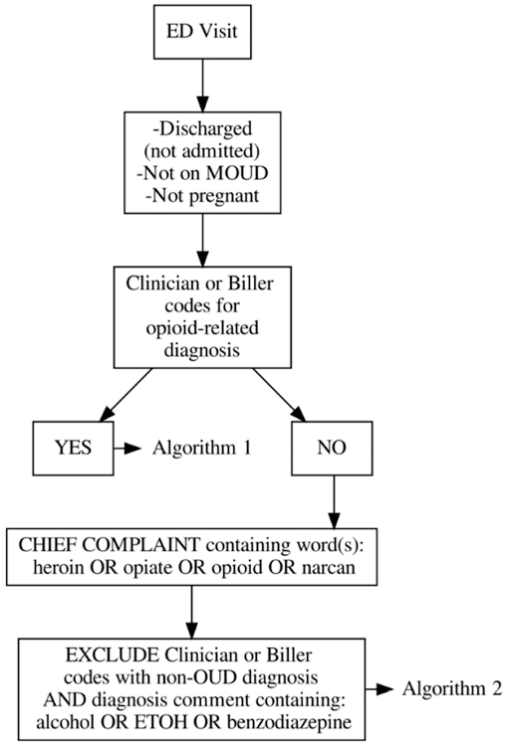
Flow diagram of the ED OUD EHR-based computable phenotype. Reprinted from JMIR [22] with permission of the authors.

A two-algorithm computable phenotype was derived and validated (internally and externally, using physician chart review as a reference standard) to identify eligible ED patients with structured data elements in the EHR suggesting the presence of OUD in discharged patients who are not pregnant and not currently receiving MOUD (Figure 1). This phenotype allows us to complete study analyses using passive data extraction from the EHR. Using diagnosis codes and chief complaints from the EHR, the phenotype was able to detect ED patients with presumed OUD with high degrees of validity across two large healthcare systems. In the internal validation phase, Algorithm 1 had a positive predictive value (PPV) of 0.96 (95% CI 0.863–0.995), and a negative predictive value (NPV) of 0.98 (95% CI 0.893–0.999), and Algorithm 2 had a PPV of 0.8 (95% CI 0.593–0.932), and an NPV of 1.0 (one-sided 97.5% CI 0.863–1). In the external validation phase, the combined phenotype had a PPV of 0.95 (95% CI 0.851–0.989) and an NPV of 0.92 (95% CI 0.807–0.978). Further details of the derivation and validation of the EHR phenotype are published in the Chartash et al. article in JMIR Medical Informatics[22].

### Scalable, Automated Warm Handoff from the ED to Community Providers

In order to streamline the ED referral process in a multi-network automated opioid treatment referral program, we performed a needs assessment of community providers offering ongoing MOUD. Stakeholders emphasized a need for automated, flexible, on-demand, and protected communication between EDs and community providers of MOUD to increase patient retention across the referral cascade. Further details on the lessons learned about the MOUD referral process are reported in the July 2019 publication by Ahmed et al. in the Journal of Substance Abuse Treatment [23].

### Integrated Web Application and Automation of EHR Workflow

Given the barriers to adoption of ED-initiation of BUP and the study goal of optimizing the intervention’s usability, test users emphasized ease of EHR integration and automation of EHR workflow in the design of the CDS implementation [21]. With these goals in mind, a web-based application was created to serve as a centralized, vendor-agnostic solution with standards-based messaging and scalability across a variety of healthcare systems. In the primary EMBED health system, this integrated web application was successfully embedded into the EHR clinical workflow using Epic’s AGL framework and flowsheet functionality. However, due to limitations of current standards-based messaging, security concerns, and the need for resource-intensive local customization, secondary EMBED health systems made local, pragmatic decisions on how to build the intervention into their system. These sites developed solutions that maintained fidelity with the study intervention’s requirements: assessing for OUD and withdrawal severity and automating documentation, orders, prescriptions, referral, and discharge instructions. Full details of the IT integration process, barriers, and solutions were reported in October 2019 in Melnick et al. article in the Journal of the American Medical Informatics Association (JAMIA) Open publication [24].

### The Integrated Web Application from the User’s Perspective

**Figure 2.**
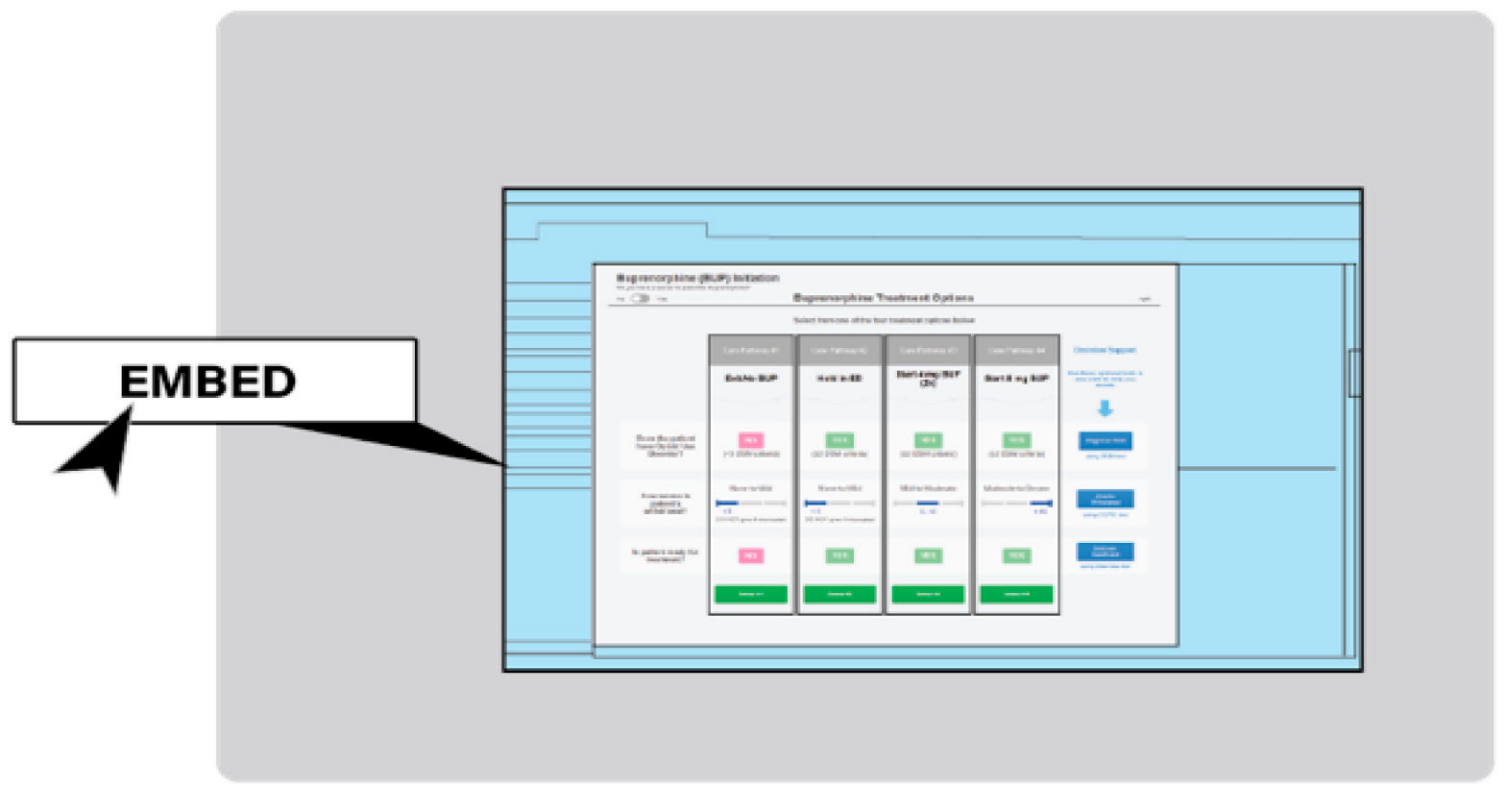
Conceptualization of EHR screen from patient chart showing the EMBED app with the button that launches it.

In the main EMBED health system, from a clinician’s perspective, the “EMBED” button can be seen in any patient’s EHR chart (Figure 2).

When the clinician clicks this button to launch the EMBED intervention, he/she is guided through the process of choosing the best care pathway with the help of three optional decision support tools which assist with: (1) assessing for OUD, (2) evaluating withdrawal severity, and (3) motivating patient readiness to begin treatment. The support system is user-initiated and offers flexibility, in terms of degree of support offered, based on the user’s needs: an experienced clinician can bypass the decision support and directly opt for the clinical pathway, while less experienced users may choose to use any combination of the three decision support modules as needed to inform the treatment pathway selection. Once a specific treatment pathway is selected, the application automatically generates relevant EHR activities including orders, prescriptions, documentation, discharge instructions, and referral. At any point during the process, the user may alter these orders to further customize patient-specific management.

### Pilot Study

A pilot study was conducted in an urban, academic ED from April-August 2018 (pre-implementation phase) and April–August 2019 (post-implementation) to study the effect of the intervention on adult ED patients identified by the study phenotype representing patients who might benefit from BUP treatment. The rate of BUP initiation increased from 2.8% (8/288) in the pre-implementation phase to 7.0% (21/298) in the post-implementation phase (*p* = 0.02). A manuscript reporting the full details of the pilot study findings is currently under review.

### Trial Launched

All systems were assessed for readiness for trial launch using a comprehensive checklist of essential milestones (e.g., IRB reliance agreements in place, intervention live with fidelity at all intervention sites, patient and provider information posted in all study sites, etc.). To avoid patient identification based on date of ED service, all 5 healthcare systems were instructed to select a trial launch date within a two-week window. Enrollment with ongoing data collection, coordination, and management for the trial is underway per the trial protocol [20]. Data collection and submission will continue monthly for the duration of the trial over the next 18 months.

## DISCUSSION

In the ongoing EMBED trial, a CDS intervention for ED initiation of BUP for OUD has been formatively evaluated with user-centered design and implemented across multiple healthcare systems. The intervention supports emergency clinicians in the management of adult ED patients with OUD who could benefit from MOUD, offering clinicians flexible, user-initiated decision support tools and launches care pathways that automate multiple EHR activities. Preliminary results for both primary and secondary outcomes in the pilot study indicate that the intervention is associated with increased adoption of ED BUP initiation. The subsequent pragmatic trial has now launched across 20 EDs in 5 healthcare systems to test the intervention’s effectiveness to increase adoption of ED initiation of BUP into routine emergency care.

The EMBED project was proposed to address the critical barriers to adoption of this evidence-based best practice. We identified barriers at the physician- and healthcare system-levels and designed the study intervention to address these barriers. Three important concerns impact clinician uptake of BUP initiation in the ED: lack of knowledge about MOUD, regulatory barriers to provision of MOUD, and the clerical burden of implementing the orders, prescriptions, and follow-up associated with BUP initiation.

The EMBED intervention addresses clinician knowledge deficits pertaining to OUD treatment by providing user-centered CDS. A 2019 survey of ED clinicians at two urban, academic EDs found that 39% of physicians self-rated themselves as prepared to determine the level of care needed by an OUD patient, while 29% felt prepared to connect OUD patients with outpatient treatment. Of all surveyed components of emergency management of OUD, emergency physicians felt least prepared to initiate BUP, with only 27% self-reporting themselves as prepared [18]. Addressing concerns over CDS implementation, our intervention delivers the right information, to the right person, in the right format, at the right time in a way that is least disruptive to the clinician and optimizes clinical decision making and clinician efficiency [25]. This is highlighted by the notion that identifying a ‘one size fits all’ type of solution cannot serve varied user needs. The EMBED intervention offers CDS in a flexible format [21].

The regulatory barriers to provision of MOUD are complex and often misunderstood. Treatment of OUD is heavily regulated, particularly the use of methadone, which generally must be provided to patients under supervision, requiring daily visits to an OTP clinic. However, the DATA 2000 Act provides a program allowing physicians who have completed 8 h of training to obtain a waiver (called an X-waiver) authorizing them to prescribe BUP in settings other than OTPs. ED clinicians, like any other clinicians with a DEA number, are eligible to obtain a DATA 2000 waiver, and can then prescribe BUP to last until patients can meet with a community provider for long-term treatment. In addition, federal regulation referred to as the “72-hour rule” (Title 21, Code of Federal Regulations, Part 1306.07(b)) allows any physician, regardless of whether they have an X-waiver, to administer BUP for up to 72 hours. The 72-hour rule can act as a bridge to upcoming treatment when no provider with an X-waiver is available to prescribe BUP for home use. The EMBED intervention includes separate treatment pathways for clinicians with and without an X-waiver to provide BUP compliant with the 72-hour-rule, offering the flexibility for any clinician to use the CDS irrespective of their DATA 2000 waiver status.

The EMBED intervention streamlines and automates the ED BUP initiation workflow, reducing clerical burden that may discourage emergency clinicians from initiating BUP. In the planning phase of the EMBED project, we conducted observation studies and interviewed clinicians and other stakeholders to identify and address key barriers in streamlining the workflow for BUP protocol [21,23]. The intervention both supports clinical decision making by guiding clinicians through assessing patients’ OUD and withdrawal symptoms and readiness for treatment, then automates routine EHR activity following a treatment choice, including note writing, prescriptions, order entry, discharge instructions, and facilitation of hand-off to follow-up with community OUD treatment providers. In this way, the EMBED intervention is designed to reduce the clerical burden associated with BUP initiation.

At the healthsystem level, the integration of a new, complicated clinical pathway into the existing EHR can be a barrier to adoption. The EMBED intervention was designed to be integrated with the majority of commonly used EHR platforms [24]. The EMBED trial includes health systems using customized implementations of two major EHR vendors’ products to test the interventions scalability and generalizability.

### Study Strengths

EMBED is a pragmatic implementation trial, designed to reduce the gap between real-world clinical practice and traditional RCTs that often include highly selected study populations and use extensive, costly supportive services. The EMBED CDS intervention does not require any additional personnel in the ED, and is being studied across diverse patient and clinical settings. The study sites include both community and academic sites, and the analysis includes all attending physicians practicing at these sites--not just those who have opted in to participate in a randomized trial. This trial is directed at reducing any negative impact on providers and increasing the likelihood of immediate impact on actual care delivery for OUD patients. Implementation of user-centered CDS will increase the likelihood of adoption of ED-initiated BUP in people with OUD, thereby increasing access to treatment options for this chronic and relapsing medical condition that carries a substantial morbidity and mortality risk within 12 months of the ED visit, with major impact on healthcare use, costs, and society.

From a clinical perspective, adopting this evidence-based practice into routine care would shift the clinical practice paradigm towards early identification and treatment of OUD by initiating treatment at a time when the patient remains motivated and particularly vulnerable to morbidity and mortality. Initiating treatment in the ED, essentially, gives the opportunity to save a life twice (e.g., resuscitation from an overdose and prevention of future overdoses in OUD patients at highest risk of overdose). For most clinical scenarios, ED protocol typically involves identification, risk stratification, treatment, and referral. Somehow, with OUD we are yet to get there. Thus, with this project, we propose a similar approach for OUD, echoing the former Surgeon General Murthy’s call to American physicians to treat OUD as a chronic illness, and not a moral failing [26].

Our approach to the design of this study was to address the major barriers that have discouraged widespread adoption of BUP initiation in the ED. While the use of BUP in OUD has been around for several years now, ED initiated BUP has not yet been adopted in routine emergency care. This delay in adoption of evidence-based practice is not unique. On average, it takes 17 years from discovery to the adoption of evidence-based practices into routine care [27]. With promising results in the pilot study, we anticipate that the EMBED CDS with its user centered design might support the large-scale adoption of ED-initiated BUP and improve outcomes for OUD patients across the country. For widespread implementation to occur across different EHR vendor products, from a technical standpoint it will be necessary for data standards to continue to mature or to explore alternative more scalable approaches to implementation such as including the intervention as a native tool in the EHR or on already scaled and widely used platforms commonly used by clinicians [24]. Also, given the stigma associated with OUD and associated low rates of treatment, future implementation efforts could work to systematically address stigma as a barrier to treatment, for example, by employing strategies that address specific dimensions of stigma [28].

### Limitations

Pragmatic trials necessarily trade off some degree of strength of causal inference in comparison with studies that require more rigorously controlled study procedures. In the case of the EMBED trial, we know that participating sites are likely to pursue clinical quality improvement (CQI) initiatives addressing the emergency care of patients with OUD. We recognize that such CQI initiatives may have some impact on our outcomes. We had initially proposed using a stepped-wedge study design where each site would serve as its own control, comparing pre-intervention and post-intervention results. However, as we progressed in the planning phase, we began to recognize major shifts in ED OUD management nationwide due to the urgency of the opioid crisis and were concerned that temporal trends from national and local initiatives to stem the opioid crisis might obscure the effect size of the EMBED intervention. Therefore, we replaced the stepped-wedge design with a group, parallel, cluster-randomized design. The group randomized design allows for a shorter study period and better controls for temporal trends and CQI initiatives. Constrained randomization of study sites was used to balance heterogeneity between sites randomized to receive the intervention. Furthermore, we are tracking CQI initiatives before and during the trial in all study sites, so we can better control for the effects of unanticipated CQI interventions. We believe these pragmatic approaches may increase the generalizability of our findings, as the study sites are not artificially constrained from their regular activities and approaches to improving clinical care.

A second limitation of this study is that it doesn’t directly address clinician attitudes toward people with OUD and the use of MOUD. Like many Americans, some ED physicians have misconceptions about OUD--considering opioid addiction as a moral failing, believing that treatment doesn’t work, that patients don’t want treatment, or that initiating BUP takes too long and is too hard to do in the ED [8]. While we would encourage all clinicians to educate themselves about the realities of OUD--that MOUD is a highly effective treatment for a chronic relapsing disease, that many people who are not yet ready for treatment benefit from compassionate, non-judgemental care in the ED--the EMBED study does not include an education component. Still, we believe that once treatment is available in the ED, and once they see that the EMBED CDS tools support a BUP initiation process that fits into the ED workflow, physicians will begin to change their minds about treating people with OUD.

## CONCLUSIONS

Patients presenting to the ED with non-fatal opioid overdose face a high risk of mortality following ED discharge but, given high-quality studies showing the life-saving impact of MOUD treatment, ED visits can provide an unparalleled opportunity for intervention to impact the outcome of this vulnerable population. Continuing to offer only symptomatic relief with advice to follow up elsewhere in the community represents a grave missed opportunity to provide safe, appropriate, patient-centered care. With promising results from the pilot phase of this ongoing multi-center pragmatic trial, we anticipate findings from the trial will provide compelling evidence of the effectiveness of a user-centered CDS intervention to improve rates of ED-initiated BUP for care of patients with OUD, and help accelerate the clinical practice paradigm shift towards early identification and treatment of OUD.
